# Influence of High *Eimeria tenella* Immunization Dosages on Total Oocyst Output and Specific Antibodies Recognition Response in Hybrid Pullets (*Gallus gallus*)—A Pilot Study

**DOI:** 10.3390/antib14010009

**Published:** 2025-01-26

**Authors:** Marco A. Juarez-Estrada, Guillermo Tellez-Isaias, Víctor M. Petrone-Garcia, Amanda Gayosso-Vazquez, Xochitl Hernandez-Velasco, Rogelio A. Alonso-Morales

**Affiliations:** 1 Departamento de Medicina y Zootecnia de Aves, Facultad de Medicina Veterinaria y Zootecnia, Universidad Nacional Autónoma de México, Cd. Universitaria, Ciudad de Mexico 04510, Mexico; xochitlh@fmvz.unam.mx; 2 Departamento de Genética y Bioestadística, Facultad de Medicina Veterinaria y Zootecnia, Universidad Nacional Autónoma de México, Cd. Universitaria, Ciudad de Mexico 04510, Mexico; amandagv66@hotmail.com; 3 Department of Poultry Science, University of Arkansas, Fayetteville, AR 72701, USA; gtellez@uark.edu; 4 Centro de Educación Agropecuaria (CEA), Facultad de Estudios Superiores Cuautitlán (FESC), Universidad Nacional Autónoma de México (UNAM), Cuautitlán Izcalli 54714, Mexico; vmpetrone@hotmail.com

**Keywords:** avian immunology, B-cell epitopes, coccidiosis vaccine, crowding effect, gut-associated lymphoid tissue, passive immunization, Immunoglobulin Y, Western blot

## Abstract

Background: Two high primary-immunization doses of a wild-type *E. tenella* strain were assessed in healthy pullets (5K *versus* 10K sporulated oocysts/bird) to understand the effects of coccidia infection. Methods: Acquired immunity was evaluated following primary immunization and two booster doses with the homologous strain. Total oocyst shedding, clinical signs, and viability of every bird/group after each immunization/booster were recorded. Indirect ELISA measured the time course of humoral responses from each immunization group against sporozoite and second-generation merozoite of *E. tenella*. Antigen pattern recognition on these two asexual zoite stages of *E. tenella* was analyzed using Western blotting with antibodies from each immunization program. Afterwards, antigen recognition of specific life-cycle stages was performed using individual pullet serums from the best immunization program. Results: A primary-immunization dose of 1 × 10^4^ oocysts/bird reduced the oocyst output; however, all pullets exhibited severe clinical signs and low specific antibodies titers, with decreased polypeptide recognition on both *E. tenella* asexual zoite stages. In contrast, immunization with 5 × 10^3^ oocysts/bird yielded the best outcomes regarding increased oocyst collection and early development of sterilizing immunity. After the first booster dosage, this group’s antisera revealed a strong pattern of specific antigen recognition on the two assayed *E. tenella* life-cycle stages. Conclusions: The *E. tenella*-specific antibodies from the 5 × 10^3^ oocysts/bird immunization program can aid in passive immunization trials and further research to identify B-cell immunoprotective antigens, which could help in the development of a genetically modified anticoccidial vaccine.

## 1. Introduction

Coccidiosis is the first and most economically significant parasitic disease afflicting chicken welfare and, consequently, the world’s poultry industry [1]. *E. tenella* is the causal agent of cecal coccidiosis, and this species is regularly isolated from broiler farms worldwide [2]. Chemoprophylaxis and live wild-type/attenuated coccidia vaccines are currently used as preventative methods [3,4].

Protective immunity can be achieved by infecting chickens with multiple low doses (trickle infections) or with a single high dosage of *Eimeria* parasites [2,4,5,6]. *E. tenella* is one of the most pathogenic strains of the *Eimeria* species that parasitize chickens; however, its true immunogenicity remains unknown [2,7,8]. While ingesting a few *E. maxima* oocysts results in 100% immunity to a homologous challenge [6,8,9], an equivalent dose of *E. tenella* oocysts fails to elicit a significant level of protective immunity [2,5,6,7,10,11]. High doses of *E. tenella* oocysts could be employed in chickens. However, the outcome regarding actual infection resistance, antibody titers obtained, and specific B-cell antigen recognition have yet to be adequately established [2,10,12,13,14,15].

The severity of illness following *Eimeria* infection is species-specific and dosage-dependent. Infection with higher amounts of sporulated oocysts leads to increased parasite burdens in the gastrointestinal tract (GIT) and elevated oocyst shedding. However, fecundity decreases as the dosage quantity increases, a reaction known as the crowding effect (CE). Estimates of maximal parasite reproduction at each life-cycle stage, host cell availability, and the CE have all been commonly used to model *Eimeria* sp. reproduction in chickens [8,14,15,16,17]. The CE is described as a decline in fertility when the number of infective parasites exceeds a given threshold. At high infection pressures, host cells may be destroyed before the subsequent life-cycle stages of the parasite are completed, limiting the number of oocysts generated. Gradually increasing oocyst doses until reaching an infection level, known as the “maximally producing dose”, eventually leads to a point where further quantity increases result in progressive declines in oocyst yields. This “crowding threshold” is the dose that gives the maximum reproductive potential, while doses exceeding it are referred to as “crowded doses” [14,15,16]. Understanding the crowding limitations of several *Eimeria* species under specific circumstances is critical for optimizing in vivo oocyst stock production for laboratory or factory vaccine settings [4,14,15,16,17].

Inflammation is a crucial event in the immunological response to chicken *Eimeria*. Many researchers have shown that the extent of inflammation, contributing to avian gut integrity damage, varies according to the *Eimeria* species, strain, vaccination schedule, dosage, litter condition, host age, nutritional status, immunological condition, and inbreeding degree of the birds [2,3,4,8,9,11,12,15,16,17]. Although the pathogenicity of *E. tenella* is evident once its life cycle is completed in terms of excreted coccidian oocysts, the extent of inflammation, and the severity of intestinal lesions, the antigenicity and immunogenicity of a primary-immunization dosage are far more difficult to quantify [2,6,8,10,11,15]. Cellular and humoral immunity are involved in the protection of chickens against *Eimeria* infections [3,5,6,8]. Several studies have shown that cellular immunity is very important, whereas the role of humoral immunity is relatively minor [2,3,5]. There is still little information regarding the pathogenic effects produced by different primary immunizing doses of *E. tenella* naturally (orally) administered to hybrid pullets regarding the structure and function of gut-associated lymphoid tissue (GALT), particularly on the early recognition of B-cell antigens of the two most antigenic asexual life-cycle stages of *E. tenella* (sporozoite and second generation of merozoite) [2,5,8,12,15,17,18]. Most published studies on this subject have compared inbred layer hen lines or young broiler chickens rather than hybrid pullets [2,5,8,11,13,14,15,19]. With the current increase in consumer awareness of laying hen welfare, the egg industry is transitioning toward cage-free production systems [1]. Pullets/laying hens are now raised in a litter, increasing the risk of clinical coccidiosis, which usually was not an issue in conventional cage systems [1,2,4,19]. We need to know more about the effects of coccidiosis on pullets before laying and its consequences [1,15,19]. A detailed analysis of the idiosyncratic hybrid pullet immune responses to B-cell-specific antigens of these biologically unique pathogens might be very illuminating. This study aims to verify the influence of two high primary immunizing dosages of *E. tenella* in pullets followed by two booster doses on total oocyst output, clinical signs, viability, and the degree of humoral immune response toward sporozoites and the 2nd generation of merozoites of *E. tenella*. The outcomes from this study could expand our comprehension of the host’s immune response to *E. tenella* and its correlation with the severity of the disease. Additionally, understanding the immunological response to primary, secondary, and tertiary infections may provide useful information for establishing successful strategies for controlling and managing *E. tenella* infections in poultry.

Furthermore, the immunoglobulin Y (IgYs) generated in response to specific *E. tenella* antigens could be a valuable resource for further studies on passive immunity strategies and identifying potentially useful candidates for developing a genetically designed *E. tenella* vaccine. In our study, using two different dosages of *E. tenella* sporulated oocysts in a priming/booster immunological program, we were able to determine the appropriate primary dosage that provides a high quantity of oocysts for laboratory stock or live vaccine manufacturing. At the same time, it allowed us to determine the critical parameters required for hybrid pullets to develop early sterilizing immunity against *E. tenella*.

## 2. Materials and Methods

### 2.1. Animals

Due to the varied nature of their reactions to *E. tenella* infection, twenty-seven one-day-old hybrid chicks (Hampshire × Rhode Island Red × Plymouth Rock Barred) were randomly selected for this study [2,3,15,19]. The birds were reared in a well-ventilated room inside a coccidia-free brooder equipped with a raised wire netting floor. The birds were provided with coccidiostat-free feed and water ad libitum. 

### 2.2. Parasites

The wild-type strain of chicken *Eimeria* used in these investigations was collected from chickens with clinical signs of cecal coccidia in a Mexican broiler farm. This wild-type *E. tenella* strain was isolated from a single oocyst recovered from an *Eimeria* sp. sample obtained from the aforementioned clinical case. It was also differentiated based on oocyst morphology, colonization site, and pathology generated. The *E. tenella* oocyst was isolated by employing the agar plate method. Briefly, this method involved cutting 5% gelatin with a scalpel blade to obtain small cuboids (4 mm × 4 mm) and then pouring 0.5% agar decrescent dilutions of a suspension of clean oocysts with a final count of 100/mL over these same cuboids of gelatin. Under microscopic control, a small block containing individual sporulated oocysts was cut from the gelatin and placed in a commercial gelatin capsule. The capsule was placed in the crop of 14-day-old SPF Leghorn chickens according to the methodology described by Stephan et al. [20]. Feces was collected six days post-inoculation and examined to confirm the presence of oocysts, while intestinal contents were obtained seven days post-inoculation.

Additionally, some technical modifications were made based on the method described by Khalafalla and Daugschies [21]. These modifications included changes to the physical support for the gelatin cuboids, the percentage of gelatin used, and the specific physical oocyst isolation technique carried out with a Pasteur pipette. This isolation was based on a single oocyst matching specific morphometric characteristics for the oocyst of *E. tenella*, as described by Haug et al. [22]. The microscopic examination of single oocyst isolates showed morphological homogeneity of the oocysts. Our wild-type *E. tenella* strain was identified using the methodology described by Fernandez et al. [23] and Ogedengbe et al. [24], which involves a multiplex PCR test based on species-specific RAPD-SCAR markers. All oocyst-related procedures, including the separation of feces, sporulation, and storage of *E. tenella* oocysts, were carried out as previously described [25,26]. The preparations of infectious dosages and quantification of oocysts shedding in feces were carried out according to the method described earlier [25,26]. The parasites were replicated by passing through SPF White Leghorn chickens, and oocysts were harvested and used within one month of sporulation [25,26].

### 2.3. Experimental Design

**Figure 1:**
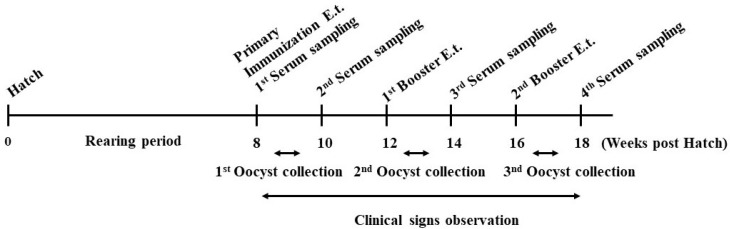
Schematic outline of the experimental design. Nine pullets were primarily immunized with either 5 × 10^3^ sporulated oocysts of *E. tenella* per bird or 1 × 10^4^ sporulated oocysts of *E. tenella* per bird at the eighth week of age. They were then boosted with the same *E. tenella* strain at 4 and 8 weeks post-primary immunization. A control group of nine pullets was maintained without any immunization or booster. Serological samples were obtained at 10, 14, and 18 weeks of age. Clinical signs were individually assessed between 8 and 18 weeks post-primary immunization, and fecal samples were collected between 5 and 9 days after every priming/booster dosage.

Twenty-seven birds were randomly assigned to three groups, each with three replicates (n = 3 birds/replicate). The first group of birds received a primary immunization of 5 × 10^3^ sporulated oocysts per bird (5K), the second group was primarily immunized with 1 × 10^4^ sporulated oocysts per bird (10K), and the third group was the unimmunized-uninfected (UU) control group (Figure 1). The quantity of sporulated oocysts in each immunization dose for each immunized group was selected based on the results shown in previous research [2,10,12,15,26]. The primary immunization dosages were administered naturally (orally) to every pullet bird at the age of eight weeks (Figure 1). Birds in both immunized groups were boosted with 1 × 10^5^ sporulated oocysts per bird four weeks following primary immunization at 12 weeks of age. All pullets in both immunized groups received a second booster (1 × 10^6^ oocysts per bird) at 16 weeks of age. A poultry veterinarian (M.A.J.-E.) recorded clinical signs of cecal coccidiosis in each immunized group daily during the prepatent and patent periods (Figure 1). Throughout the experiment, the UU control birds were maintained in an adjacent unit under specific pathogen-free and controlled environmental conditions.

### 2.4. Clinical-Sign-Based Rating Score

The clinical signs of cecal coccidiosis in each bird of the immunized and unimmunized groups were monitored daily through observation. Data from each group were recorded during the prepatent and patent periods of primary *E. tenella* infection. A clinical rating scale was elaborated based on the poultry’s main clinical signs of cecal coccidiosis [8,11,17]. This rating scale included the following seven traits: lethargy, prostration, ruffled feathers, loss of appetite, dehydration, bloody feces, and watery droppings [11,17,27,28]. Every clinical manifestation received a rating score, as follows: no clinical sign = 0, slight = 1, mild = 2, severe = 3, and highly severe = 4. The clinical sign rating score for each group was defined as the sum of all pullet scores for each clinical sign grading.

### 2.5. Oocyst Harvesting and Serum Samples

Oocyst excretion was measured in each immunized group from 5 to 9 days post-inoculation (PI) [25,26] (Figure 1). The total oocyst output was calculated as the number of oocysts excreted by every bird in each experimental group [26]. Negative control birds (UU) were screened for oocysts per gram of feces (OPG) throughout the experiment. All birds were wing-bled at the first immunization date (preimmunized sera) and two weeks after every priming/booster dose. Serological samples were obtained at 8, 10, 14, and 18 weeks of age (Figure 1). At the same time, blood samples were collected from the UU control group pullets. The serum was separated by centrifugation at 2000× *g* for 5 min, and the samples were frozen and kept at −20 °C until use.

### 2.6. Preparation of Antigens from Sporozoites and Second-Generation Merozoites of Eimeria tenella

The *E. tenella* sporozoites (Sz) were purified using the method described by Constantinoiu et al. [29]. Briefly, the sporocysts were released by vortexed sporulated oocysts (2.5 × 10^7^/mL) at 2000× *g* using 1 mm diameter glass beads (Sigma-Aldrich, Inc., Burlington, MA, USA) for 1 min. A 50% Percoll gradient (density 1.13 g/mL, GE Healthcare, Piscataway, NJ, USA) was used to purify the sporocysts [29]. The purified sporocysts were resuspended in an excystation medium and incubated at 42 °C for 150 min. The excystation medium consisted of PBS (pH 7.4) with 0.75% (*w*/*v*) taurodeoxycholic acid (Sigma-Aldrich, Inc., Burlington, MA, USA) and 0.25% (*w*/*v*) trypsin from porcine pancreas Type II-S (Sigma-Aldrich, Inc., Burlington, MA, USA). The sporozoites (Sz) were washed and purified using a 60% Percoll gradient (density 1.13 g/mL, GE Healthcare, Piscataway, NJ, USA) [30]. The second-generation *E. tenella* merozoites (Mz) were purified according to the methodology described by Constantinoiu et al. [29,31]. Briefly, three 10-week-old hybrid pullets were gavage-inoculated with 5 × 10^5^ sporulated oocysts of *E. tenella* and euthanized 112 h post-infection (PI) to collect second-generation merozoites. The intestines of these birds were treated for merozoite isolation, as described previously by Liu et al. [32]. The second-generation merozoites were purified using the method outlined by Geysen et al. [33].

Purified parasites (Sz and Mz) for antigen preparation were resuspended in sterile PBS with protease inhibitor (cOmpleteTM Roche Applied Science, Mannheim, Germany), gradually frozen at −70 °C at a rate of 1 °C/min, and then stored at −70 °C until use.

Five freezing and thawing cycles were performed repeatedly to disrupt the parasites. The final suspension was centrifuged at 2000× *g* for 18 min at 4 °C. The supernatants were collected, and the protein concentration was determined using the Bradford reagent (BioRad, Hercules, CA, USA) with a bovine serum albumin standard curve (Sigma-Aldrich, Inc., Burlington, MA, USA), according to the manufacturer’s directions. Antigen suspensions were kept in 200 μL aliquots at −70 °C until needed.

### 2.7. ELISA Measuring IgYs

Antisera reactivity to Sz and Mz antigens was assessed by indirect ELISA, as previously described by Juárez-Estrada et al. [34]. Briefly, 96-well microtiter plates (MaxiSorb, Nunc, Roskilde, Denmark) were coated overnight at 4 °C with 1 μg of sporozoite or 2nd-generation merozoite antigen in 100 μL of carbonate buffer (0.1 M sodium bicarbonate and 0.1 M sodium carbonate buffer, pH 9.6). Control wells were incubated with 100 μL of carbonate buffer. After four washes on a shaker with a saline solution (S) (120 mM NaCl, 25 mM Tris-HCl, pH 7.9) containing 1% Tween 20 (ST), non-specific binding sites were blocked by incubating for 1 h at 37 °C in a static oven with 110 μL of 5% skim milk in ST (STM). Serum diluted 1:100 in STM was added to the test and control wells after four ST washes, and both were then incubated for 1 h at 37 °C. Each plate included negative control serum samples from unimmunized-uninfected pullets. After incubation, the plates were washed four times with ST and then incubated with their respective secondary antibody peroxidase conjugate diluted 1:2000 with STM (Rabbit Anti-Chicken IgY IgG) (Jackson ImmunoResearch Laboratories, Inc., West Grove, PA, USA). Following 1 h at 37 °C, the plates were washed four times. The enzymatic reaction was developed by adding 100 μL of OPD chromogen (o-phenylendiamine dihydrochloride SIGMA, St. Louis, MO, USA) at 5 μg/10 mL in citrate buffer (0.1 M citric acid, 0.1 M sodium citrate p.H. 4.5, and 20 μL of 30% hydrogen peroxide) for 10 min on a shaker in the dark. An ELISA microplate spectrophotometer (Epoch, BioTek, Winooski, VT, USA) was used to measure the absorbance produced by substrate hydrolysis at 450 nm.

Positive control sera were obtained from four 12-week-old SPF White Leghorn chickens subcutaneously vaccinated with 5.3 × 10^6^ whole-sporozoites of *E. tenella*. The chickens were boosted thrice with the same dosage at 2-week intervals [34]. All serum samples were evaluated in duplicate and examined twice to identify outliers.

### 2.8. SDS-PAGE and Western Blotting

Under reducing conditions, 12% SDS-PAGE was used to separate 20 μg of each purified fraction of the two *E. tenella* life-cycle stages [34]. The resolved proteins were either stained with Coomassie Brilliant Blue (CBB) or transferred electrically onto a polyvinylidene difluoride membrane (PVDF, Bio-Rad, Hercules, CA, USA). PVDF membranes were probed with a pool of six anti-*Eimeria tenella* serums collected randomly from each immunized and unimmunized group of birds.

After the second booster, three antiserums from Group 5K were randomly selected and individually analyzed (n = 3) using ELISA before being analyzed with Western blotting (WB). All serum samples were diluted at a ratio of 1:100. For the secondary antibody, horseradish peroxidase (HRP)-conjugated IgG goat anti-chicken IgY (Jackson ImmunoResearch Laboratories, Philadelphia, PA, USA) was used at a concentration of 1:1500. The PVDF membranes were visualized using DAB tablets of 3,3′-diaminobenzidine (10 mg/5 mL) (SigmaFast™, Sigma-Aldrich Corp., St Louis, MO, USA). All bands detected in the Western blotting trials were analyzed by measuring their intensity after background subtraction of the unimmunized birds’ sera control images using the ImageJ.JS ImJoy analysis tool. Available online: https://imagej.net/ij/index.html (accessed on 13 October 2023). 

### 2.9. Parameters to Assess Acquired Immunity

The severity of the clinical signs in birds of both immunized groups was compared using an ordinal-scale system based on the main coccidia pathognomonic clinical signs during the prepatency (time interval between parasite entry into the host and the appearance of oocysts in feces) and patency (time interval between the beginning of oocyst output by the birds and cessation of the infection) [11,17,27,28]. At each sampling interval, the time course of the antibody response to Sz and Mz antigens in the immunized and control birds was compared. To investigate the differences in total OPG per bird by group, the birds in the immunized and control groups were compared after primary immunization and after each booster, with every pullet functioning as the experimental unit.

### 2.10. Statistical Analysis

Using a general linear model (GLM) module, the data were collected and subjected to ANOVA. The UNIVARIATE procedure of SAS/STAT 9.2 data editor software (SAS/STAT 9.2. SAS Institute Inc., Cary, NC, USA) and Bartley’s test were used to evaluate the normal distribution of residuals and variance homogeneity of the data, respectively [21]. When the overall analysis revealed differences between the groups, a post hoc multiple comparison analysis (Tukey’s test) was used to distinguish significant differences between the means, with a 5% degree of significance. The effect of each primary dosage on the clinical signs rating score was determined using a nonparametric one-way ANOVA procedure, with significance assessed at *p* < 0.05. The blots of immunodominant-specific antigens from both asexual zoite stages of *E. tenella* were assessed by two independent observers to discriminate singularities and concatenate the molecular mass of each detected band.

## 3. Results

### 3.1. Clinical Signs and Mortality

**Figure 2:**
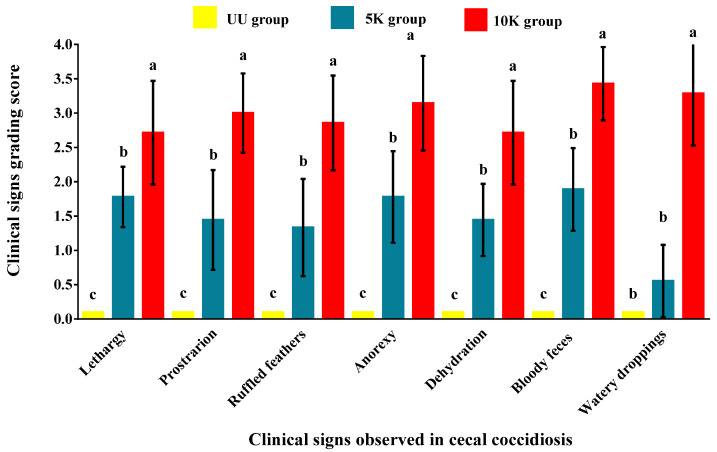
Clinical signs grading scores were recorded during the prepatent and patent periods after primary immunization of hybrid pullets at 8 weeks of age either with 5000 or 10,000 sporulated oocysts of *Eimeria tenella.* An unimmunized-uninfected (UU) control group was included in this study. The bars indicate median values for each treatment group. Median points are displayed in the figure represented by the arithmetic mean (±SD) in order to achieve the best comprehension of the visualized data. Different letters (a, b, and c) next to each bar indicate statistical differences (*p* < 0.05). The 5K and UU groups (n = 9), and 10K group (n = 7).

The main clinical signs (lethargy, prostration, ruffled feathers, anorexia, dehydration, bloody feces, and watery droppings) were noted five days post-inoculation in the birds immunized with both primary dosages of E. tenella sporulated oocysts. Mean clinical sign scores were significantly higher (*p* < 0.05) in the 10K group than in 5K group (Figure 2). During the prepatent period, the pullets that received 10K sporulated oocysts as primary immunization showed more severe clinical signs than the birds primed with 5K sporulated oocysts. Two pullets from the 10K group (22%) died on day 6 PI. Both ceca were processed [21], and a mean of 1.66 × 10^7^ oocysts per bird was quantified.

Interestingly, although all surviving birds from the 10K group showed complete recovery following the patency period (~14 days PI), they displayed severe shedding of watery droppings during the remaining part of this study. Throughout the experiment, disposing of the watery feces was a major issue. All trays below these birds’ wire netting floor cages collected excessive watery droppings, making proper waste disposal difficult. There has been little to no published research on this type of clinical consequence following *E. tenella* immunization trials. The pullet birds immunized with 5K sporulated oocysts showed less severe clinical signs (Figure 2), including a hunched posture, ruffled feathers, and watery and bloody diarrhea. However, none of the birds in the 5K group died, and all birds recovered completely by the end of the first patency period (~14 days PI). The UU control group showed no clinical signs of coccidiosis or avian disease (Figure 2).

### 3.2. Identification of the Eimeria tenella Wild-Type Strain

**Figure 3:**
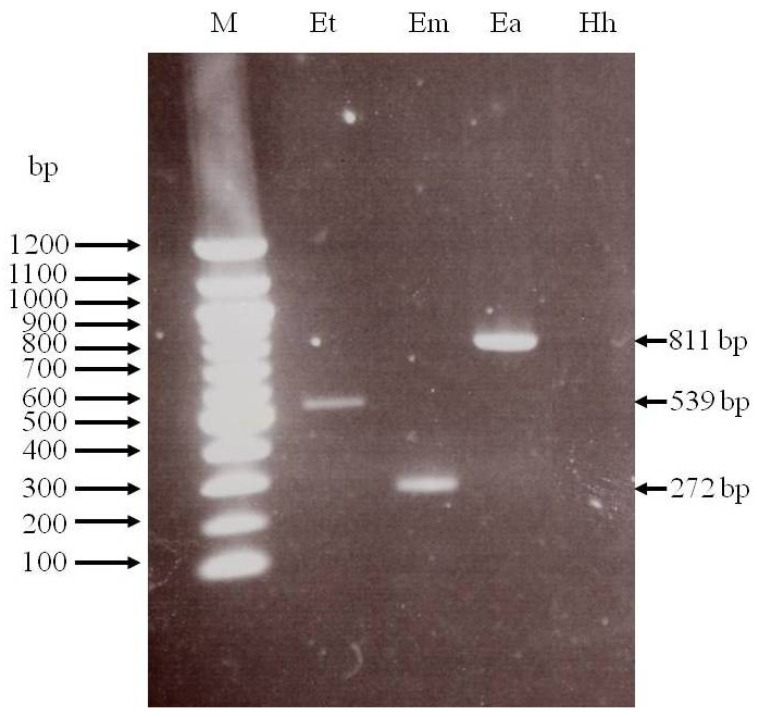
Agarose gel electrophoresis of RAPD-SCAR-based multiplex PCR products using DNA samples of the wild-type *E. tenella* strain (lane Et), *E. maxima* (M6 strain, lane Em), and *E. acervulina* (Guelph strain, lane Ea) as templates. The expected size for every amplicon is indicated on the right. Specific markers for *E. tenella* were tested with DNA of *Hammondia heydorni* as a negative control (lane Hh). Molecular size markers (lane M) in base pairs (bp) are indicated on the left.

DNA samples from purified wild-type *E. tenella* oocysts were tested with the specific primer pair in the multiplex PCR trial. The tested wild-type *E. tenella* strain was detected and presented an amplification band of the expected size (539 bp) (Figure 3). No interspecies cross-reactivity was observed with any other strain used as a control, corroborating the species-specific nature of the RAPD-SCAR markers used.

### 3.3. Oocyst Output

**Figure 4:**
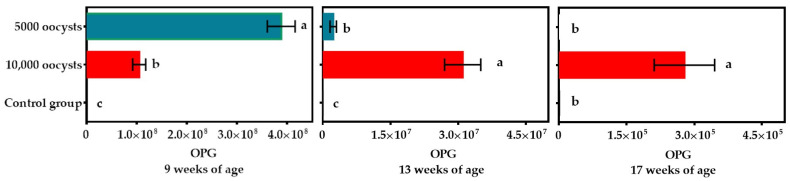
Oocyst output by pullet after primary immunization at 8 weeks of age either with 5000 or 10,000 sporulated oocysts of *Eimeria tenella*. Both groups were subsequently twice boosted with the homologous strain. The bars indicate mean values ± SD of oocysts per gram of feces (OPG). Treatments are indicated on the left. Different letters (a, b, and c) next to each bar indicate statistical differences (*p* < 0.05). The 5K and UU groups (n = 9), and 10K group (n = 7).

At nine weeks of age, the amount of oocysts shedding from the 5K group was significantly (*p* < 0.05) higher (1.30 ± 0.16 × 10^8^/bird) than the oocyst output recorded in the 10K group (0.35 ± 0.02 × 10^8^/bird) (Figure 4). At 17 weeks of age, after two boosters with the homologous *E. tenella* strain, the oocysts shedding in the 5K group stopped completely, while the oocyst output in the surviving birds of the group immunized with 10K oocysts (2.78 ± 0.87 × 10^5^/bird) was still ongoing (Figure 4).

### 3.4. Time Course of Serum Antibodies Response

**Figure 5:**
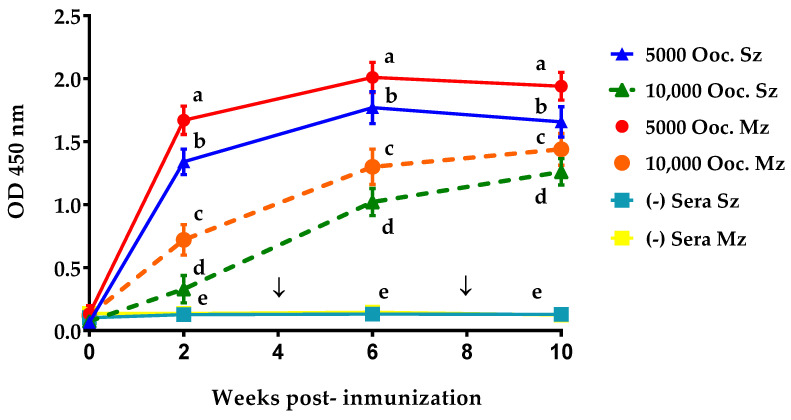
Comparison of the time course of the antibody response to sporozoite (Sz) and merozoite (Mz) antigens in birds orally primed with 5000 or 10,000 oocysts of *E. tenella* at eight weeks of age. The response to both antigens from unimmunized control birds (-) is also shown. The arrows indicate the time of boosted infections (↓). At each blood sampling, the arithmetic means (±SD) of optical densities of serums (1:100) collected from all birds in each group are shown (n = 9). Line plots with different letters (a, b, c, d, or e) within the graph indicate significant differences at *p* < 0.05.

Figure 5 depicts the kinetics of the antibody response to sporozoite and merozoite antigens following immunization and two subsequent boosters with *E. tenella* in each experimental group. At two weeks post-immunization, both experimental groups’ antibody responses demonstrated higher reactivity to second-generation merozoites than to sporozoites. Our results have revealed that the antibody response in the 5K group was faster and more robust (*p* < 0.05) than that observed in the 10K group (Figure 5). The 5K antibodies titer increased two weeks after the first booster, reaching a plateau at eight weeks PI. After the second booster, the antibody level did not rise further (Figure 5). A similar serological pattern was observed in the 10K group, although at a lower antibody level, indicating that no maximum peak or a plateau was reached. Throughout the experimental trial, there was no increase in antibody levels against the two asexual zoite stages of *E. tenella* in the sera of the UU chickens kept in *Eimeria*-free conditions (Figure 5).

### 3.5. SDS-PAGE of Sporozoites and Merozoites of 2nd Generation of E. tenella Proteins

**Figure 6:**
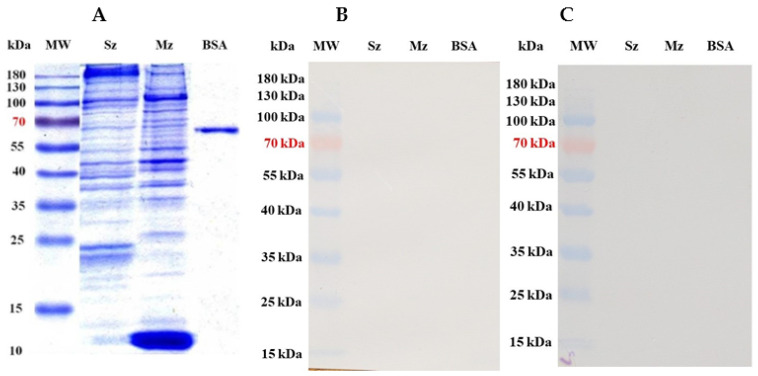
Proteins from the sporozoite (Sz) and second-generation merozoite (Mz) of the wild-type *E. tenella* strain were resolved on 12% SDS-PAGE and stained with Coomassie Brilliant Blue (**A**). The pooled sera (n = 6) of preimmunize (**B**) and unimmunized-uninfected control birds (**C**) did not show any reactivity against either of the asexual zoite stages of *E. tenella* (Mw = protein molecular weight marker; BSA = bovine serum albumin).

Figure 6A shows the polypeptide patterns of both *E. tenella* asexual zoite stages separated on 12% SDS-PAGE. Despite a complicated protein pattern, significant differences between sporozoites and second-generation merozoites were found, mainly below 50 kDa. High-molecular-weight proteins were identified in both stages, but in different quantities (>Mz). The sporozoites showed staining bands of approximately 185, 105, 94, 47, 39, and 37 kDa, as well as two bands close to 25 kDa. The merozoites of the second generation displayed significant bands at 105, 53, 47, 42, 37, 26, and 12 kDa. The pooled pre-immunization sera from the immunized birds (Figure 6B) and the pooled sera from the unimmunized-uninfected control birds (Figure 6C) did not show any reactivity against either of the asexual zoite stages of *E. tenella*.

### 3.6. Antigenic Recognition by Antisera from Immunized and Boosted Pullets

**Figure 7:**
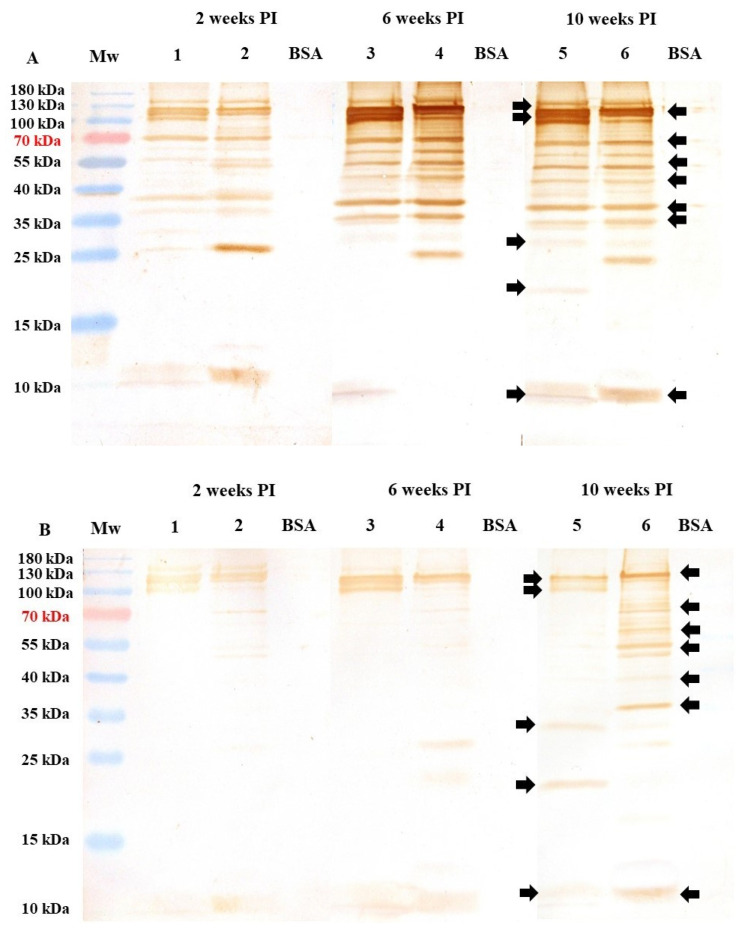
Dynamics of antigenic recognition patterns of sporozoite and merozoite of *E. tenella* by pooled antisera (n = 6) from birds immunized with 5000 (**A**) or 10,000 oocysts (**B**) and two subsequent boosters. Sporozoite (Sz) and merozoite (Mz) supernatants were used as immunoprobes every 2 weeks post-immunization (PI). Pooled antisera were diluted 1/100. Pooled sera (n = 6) of preimmunize and unimmunized-uninfected control birds did not show any reactivity against either of the asexual zoite stages of *E. tenella* (Figure 6B and Figure 6C, respectively). The black arrows show the same polypeptides on both asexual zoite stages of *E. tenella* recognized by the pullets independently of the immunization program applied. Mw = protein molecular weight marker; BSA = bovine serum albumin. Sporozoite and merozoite bands identified in both immunization schedules correlate with bands of the CBB stained 12% SDS-PAGE of Figure 6A.

Each immunization schedule was assessed using immunoblotting to evaluate specific immune responses to sporozoite and second-generation merozoite antigens (Figure 7). We discovered that the immunized birds had cross-reacting antibodies to either the sporozoites or 2nd generation of merozoites. The main difference between both groups was the higher antigen reactivity displayed by the 5K group, consistent with the stronger OD reactivity shown by this group in the ELISA test (Figure 5). Another remarkable difference is that the 5K group’s antisera recognized several high-molecular-weight antigens, common in sporozoite and merozoite proteins. After the first booster, the 5K group’s antisera increased the antigen’s recognition of high molecular weight in both parasite stages. However, this reactivity did not increase further after the second booster. Antisera detected a specific merozoite antigen close to 26 kDa, a specific sporozoite antigen of 19 kDa, and a common 10–12 kDa antigen (Figure 7A).

In contrast, the antisera from the 10K group displayed a clear recognition pattern after the second booster. Several merozoite-specific antigens with high molecular weights exceeding 35 kDa were recognized by antisera from Group 10K, the majority of which were also recognized by antisera from Group 5K. In both parasite stages, antisera from Group 10K identified an antigen with a high molecular weight of around 120 kDa. This polypeptide was the most abundant in the second generation of the merozoite stage, but two specific sporozoite antigens of low molecular weight, approximately 31 and 23 kDa, were also identified (Figure 7B).

### 3.7. Serological Analysis of Three Antiserums from Pullets Primary Immunized with 5000 Oocysts

**Figure 8:**
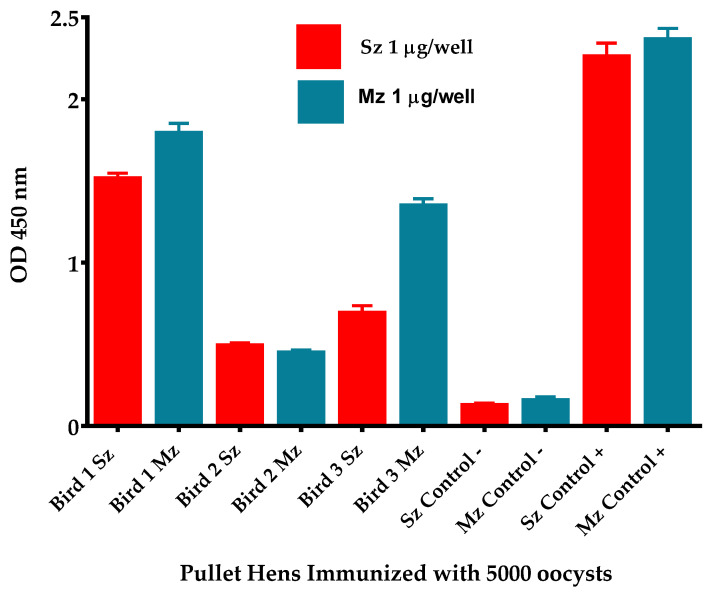
Antiserum reactogenicity to sporozoite (Sz) and merozoite (Mz) antigens of three 18-week-old pullets previously primed with 5000 oocysts of *E. tenella* followed by two boosters with high sporulated oocyst dosage. The antiserums and both reference sera (negative and positive controls) were diluted 1:100. Data are expressed as the arithmetic mean OD (±SD) of triplicates of a representative experiment.

**Figure 9:**
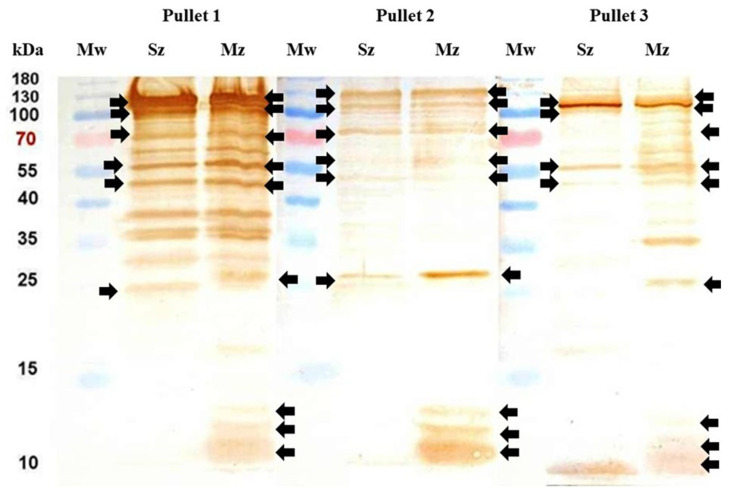
Immunoblots of sporozoites and merozoite antigens probed with serums from three pullets primary immunized with 5000 oocysts of *E. tenella* followed of two subsequent immunological boosters. Individual lanes of the Sz and Mz were loaded with 20 µg of each protein supernatant. The immunoblots were performed with individual serums from Pullet 1, Pullet 2, and Pullet 3, respectively. Each serum was diluted 1/100. After two weeks of the last booster, the black arrows show the same molecular mass polypeptides on both asexual zoite stages of *E. tenella* recognized by each pullet antiserum. Mw = protein molecular weight marker. The sporozoite and merozoite bands identified here correlate with the bands of the CBB stained 12% SDS-PAGE of Figure 6A.

Two weeks after the second booster, the immunological responses of three pullets randomly selected from the 5K group were assessed by indirect ELISA (Figure 8) and Western blotting (Figure 9). The indirect ELISA showed that the 5K antibody titer from each bird toward both antigens was higher than the sera from the UU control group. Pullet 1 displayed the highest titer, whereas Pullet 2 showed the lowest. Pullets 1 and 3 showed higher OD reactivity to second-generation merozoites than to sporozoites (Figure 8).

### 3.8. Western Blot Individual Analysis of Three Pullets with a Primary Immunization of 5000 Oocysts

Western blotting analysis of individual pullet serum from the 5K group showed heterogeneous antigenic recognition. Pullet 1 antiserum detected a broad range of total proteins observed in the SDS-PAGE-stained gels (Figure 6A). In contrast, Pullets 2 and 3 antiserums recognized only a small proportion of the total proteins (Figure 9). The antiserum from Pullet 1 detected several high molecular weight bands in both *E. tenella* life-cycle stage antigens, most of which were higher than 35 kDa, including a major one of 12 kDa in the merozoite antigen. The antiserums from Pullets 2 and 3 recognized few bands, with the majority being on the merozoite antigen. Four merozoite-specific polypeptides of ~12, ~26, ~70, and ~120 kDa were strongly identified by each antiserum. The antiserum from Pullets 1 and 3 recognized a merozoite-specific immunodominant antigen of 36 kDa. A similar polypeptide was observed in sporozoites but was only identified by the antiserum of Pullet 1. An additional 10 kDa immunodominant antigen that appeared to be unique to the sporozoite was detected by Pullet 3 antiserum. Three bands of ~53 kDa, ~56 kDa, and ~105 kDa in both asexual zoite stages were recognized by the three antiserums examined (Figure 9).

## 4. Discussion

A decrease in fecal oocyst output after challenge or booster with *Eimeria* species is an efficient technique to assess protective immunity in immunized chickens [2,11,15,34,35]. In addition to clinical protection, a vaccine that reduces oocyst shedding is desirable, as it decreases oocyst burden in the environment and diminishes the risk of infection in birds [2,3,4,6]. The 5K group initially shed more OPG after primary immunization, but after the first merogony stages, these birds could maintain enough enterocytes in the ceca. Consequently, subsequent sexual stages were able to complete their development, resulting in more shedding of OPG in the feces, which is a classical feature for a dose close to the “crowding threshold” [12,14,15,16,17]. At 17 weeks after the second booster, no oocysts were detected in the feces of Group 5K, suggesting that the birds had likely developed sterile immunity to *E. tenella* by this time.

Following primary immunization, the birds in Group 5K exhibited only mild clinical signs (Figure 2), suggesting that the parasite may have induced moderate inflammation and minor tissue damage in the intestine [2,11,12,15,17]. In contrast, the birds immunized with 10K oocysts produced significantly less oocysts following primary immunization. However, oocyst shedding indicated that the birds continued to produce a significant amount of OPG throughout the experiment (10 weeks), even after the last booster (Figure 4), which suggests that these birds developed limited immunity after primary immunization, and, as a result, they were not completely immune even after the last booster. In contrast to these findings, Riley et al. [36] found that birds immunized with 1 × 10^4^ oocysts per bird produced more oocysts than birds immunized with 5 × 10^3^ oocysts per bird.

Nevertheless, Riley et al. [36] recognized inconsistencies in their results. They identified the age of the birds as a limiting factor between achieving optimal oocyst production (for laboratory or factory setting) and an increase in mortality rate. Several research groups have shown the quantity of sporulated oocysts administered to birds in order to propagate *Eimeria* spp. oocysts varies depending on factors such as the species employed, the strain’s pathogenicity, and the study’s intended purpose, whether it be for laboratory stock, vaccine manufacturing, or priming/challenge vaccine testing [2,4,9,10,14,15,16,17,36,37]. However, the results observed in the 10K oocysts group contradict the recommendation by Holdsworth et al. [38], who suggest doses of 10^4^–10^5^ sporulated oocysts for effective propagation of *E. tenella*.

Many researchers have traditionally used high doses of coccidia (e.g., 1 × 10^4^ oocysts/bird), under the assumption that larger dosages lead to increased exposure and higher antibody titers [2,10,12,13,14,36,38]. Contrary to conventional practices, the current research has revealed that primary immunization with 1 × 10^4^ oocysts/bird of *E. tenella* led to decreased oocyst shedding and induced more severe clinical signs in birds (Figure 2). These effects were accompanied by incomplete immune protection against coccidia (there were two casualties in this group). The severity of the clinical signs was greater in the birds immunized with the highest dose of oocysts, indicating a clear intestinal barrier disruption and a large increase in intestinal permeability [17,19,28]. Therefore, the systemic effects on bird health correspond to the inflammation events occurring in the cecal tissue [12,15,17,19,27,28]. The early endogenous asexual zoite stages of *E. tenella* are generally considered more immunogenic than the later sexual stages [11,39]. If the primary immunization dose results in high infection pressure, host cells might be destroyed before the parasite’s development is completed [17], which reduces the number of oocysts produced and limits the development of a protective immune response [2,16]. Davis et al. [12] suggested that the number of lymphoid cells in the cecal submucosa directly correlates with immunoglobulin levels against *E. tenella*. However, if a dose of *E. tenella* induces early parasite crowding and depletes lymphoid cells prematurely, the remaining cells may not generate a successful protective immune response. The group immunized with 10K exhibited a classical crowding effect. This dosage corresponds to what is commonly known as the “crowded dose”. Williams [14,16] previously described the crowding effect on oocyst shedding; however, the precise mechanism by which the crowding effect influences the severity of clinical signs and mortality rate in response to increasing infection levels remains unclear [14,16,17].

Soutter et al. [15] immunized three different breeds of commercial layer chickens with high doses of *E. tenella* oocysts (Houghton strain) (4 × 10^3^, 8 × 10^3^, and 1.2 × 10^4^ oocysts/bird) without observing differences in mortality rates among the groups. Furthermore, in Soutter’s study, the different dosage sizes did not affect the lesion score, parasite replication, or cytokine transcription level at the ceca. Some of these results contradict our findings. Therefore, several factors may explain these discrepancies, as follows: (i) understanding the differences in parasite infection and mortality rates observed in our study against Soutter’s findings is dependent on the particular virulence of the strain of *E. tenella* used; (ii) differences in genetic lineage and age of the birds in each study; and (iii) potential inaccuracies in calculating the actual quantity of sporulated oocysts for each experimental immunization dosage [17,18,25,26,37]. Indeed, the freshness of the sporulated oocysts used in the inoculum could also significantly influence infection outcomes [17,37]. Tomley [37] considers the freshness grade as a major criterion when determining the number of sporulated oocysts to employ in infection trials, suggesting that the fresher the oocysts, the fewer should be used. We found that administering an initial dose of 5000 sporulated oocysts of our *E. tenella* strain to hybrid pullets significantly optimized the aforementioned objectives. However, further studies are required to address the discrepancies among factors such as the specific strain of *E. tenella*, the age and genetic lineage of the birds, the accuracy of immunization doses, the extent of intestinal damage, the clinical sign severity, and the cytokine transcription levels at GIT associated with different primary immunization dosages.

The kinetics of antibody responses in the ELISA test against sporozoite and merozoite antigen preparations showed similarity, suggesting that both life-cycle stages share multiple antigens [29,34,39,40,41]. The antibodies from Group 5K recognized both asexual zoite stages of *E. tenella* and showed a higher OD following primary immunization than those from Group 10K. Group 5K maintained higher antibody titers throughout the study period. Despite using the same quantity of each antigen (1 μg/well) in the ELISA test, Group 5K exhibited a slightly higher antibody response to the merozoite antigen compared to the sporozoite antigen (Figure 3). Our results align with Constantinoiu et al.’s [29] findings, who assayed 1 μg/well of each antigen in an experimental ELISA and found that antisera from immunized birds kept in cages and challenged twice reacted slightly more with merozoite than with sporozoite. The higher OD reactivity observed with merozoites suggests that this asexual zoite stage may be more immunogenic than sporozoites when infection occurs naturally (orally) rather than through the parenteral route [29,34]. Antibodies were detected in Group 5K two weeks after the first immunization, which then peaked six weeks later, and then declined slightly before reaching a plateau at eight weeks PI. The dynamics of the antibody response to sporozoite and merozoite antigens in *E. tenella*-infected chicks from Group 5K differed significantly from that previously described in birds immunized and reared on the floor [35,39].

Smith et al. [13] assessed the exposure of chickens to *E. tenella* and found that antibody titers against the sporozoite antigen of *E. tenella* increased only slightly after primary infection in birds immunized with 10K oocysts. Smith et al. [13] discovered that levels of sporozoite-specific IgY increased significantly after a challenge, with higher levels on day 12 post-challenge (PC) compared to immunized but unchallenged birds. Smith et al. [13] identified a humoral response similar to the antibody kinetics observed in our 10K group; however, our 5K group did not exhibit the same antibody pattern following either the first or second booster. When birds were immunized and raised on mesh without recirculating oocysts, Constantinoiu et al. [39] noted that elevated levels of specific IgYs persisted for only one week before gradually declining over the following three weeks, eventually reaching a plateau. On the other hand, specific IgY levels peaked at 3 weeks PI in chickens raised under conditions that facilitated oocyst recirculation, then gradually declined, and reached a plateau at 6–7 weeks PI. Challenge infections significantly boosted the specific IgY response in birds without oocyst recirculation. In contrast, specific IgY levels decreased immediately after the first challenge or booster in birds raised under conditions allowing free oocyst recirculation [39].

The immunization schedule employed in Group 5K closely resembled a schedule previously described by Constantinoiu et al. [39]. In this case, the birds’ pre-immune status is the decisive factor influencing the level of specific IgYs observed in each immunization schedule examined here. The group immunized with 5K exhibited greater antibody reactivity after the second booster (Mz = ~1.9 OD) than the group immunized with 10K (Mz = ~1.3 OD). Otherwise, Constantinoiu et al. [29] observed a specific IgY titer (Mz = ~1.8 OD) ten weeks following their primary immunization. Considering precautions of interpretation due to differences in laboratory reagents, poultry breed, dosage size, and *E. tenella* strain used, this titer closely resembles the IgY titer observed in our 5K group after the second booster. The antibody response pattern observed in Group 5K indicates an affinity maturation event, suggesting the development of complete humoral immunity against *E. tenella* following the first booster. Indeed, if we regard the cessation of oocyst production after a challenge or booster as a key indicator of achieving sterilizing immunity [4], our findings suggest that at least one booster dose is necessary to attain sterile immunity to coccidia. This study has demonstrated that administering a 5K immunization dose to 8-week-old hybrid pullets achieves a favorable balance between oocyst production (for laboratory stock or live vaccines) and the yield of *Eimeria tenella*-specific antibodies suitable for passive immunity in chickens, as previously described by Morales and Lucio [42] and Juárez-Estrada et al. [43].

Purified *E. tenella* sporozoites and second-generation merozoites were subjected to SDS-PAGE analysis, and the results revealed that their polypeptide profiles were just as complex as those documented previously [18,31,39,44,45,46]. Although most antigens appeared to be shared, Western blotting unveiled some antigen variations between sporozoites and second-generation merozoites. Previous studies using rabbit and avian antisera revealed that sporozoites and merozoites shared certain antigens [18,34,39,46,47]. Most antigens with molecular weights exceeding 50 kDa seemed to be shared by both life-cycle stages [31,34,39,44,47]. The immunization with 5K oocysts per bird led to the production of specific antibodies against *E. tenella*, which exhibited a distinct and clear pattern of antigen recognition immediately after primary immunization. The antisera obtained from the 5K group identified bands of different molecular weights, and the clarity of these bands improved slightly with each booster dose (Figure 7A). In contrast, the antisera from Group 10K exhibited a weak recognition pattern for antigens on both life-cycle stages, even after the final booster dose (Figure 7B). Evidently, the immunological response in Group 10K was severely impaired. Consequently, the antisera from these birds did not exhibit strong antigen recognition on either of the asexual zoite stages and only displayed a few barely detectable bands. Indeed, the antibodies from the 10K group showed weaker reactivity with the sporozoite antigen than the merozoite antigen, which suggests that infection with the initial life-cycle stage of *E. tenella* (sporozoite) led to an immune response that was inherently weak from the outset of the first immunization (Figure 7B). This shows a classic case of dose-immune impaired response, where administering a high dose of infective sporozoites (10K oocysts/bird) results in increased damage to epithelial and intraepithelial intestinal cells [11,12,14,16,17,19]. A further assay to measure the affinity level of IgYs from both groups is needed. T lymphocytes appear to react to coccidial infection by producing cytokines and directly engaging in cytotoxic attacks on infected cells [5,6,7,8,15,19]. Some studies have demonstrated that *E. tenella* sporozoites invade and destroy enterocytes and affect a significant number of lymphoid cells [3,11,13]. Dalloul and Lillehoj [3] have shown that CD8+ T-cells play a crucial role in the immunological recognition and containment of *E. tenella* during post-challenge infection stages. If the integrity and functionality of antigen-presenting cells (APCs), such as macrophages, dendritic cells, and B lymphocytes, as well as CD4+ and CD8+ T-cells, are compromised from the start of the first immunization, owing to a higher dose of *E. tenella* oocysts (referred to as the “crowded dose”), the birds may develop an ineffective immune response in subsequent infections due to a lack of competent lymphoid cells [2,7,10,11,12,14]. The birds immunized with 10K oocysts exhibited a weaker immune response, likely attributable to impairment of cellular immunity at the ceca level. The outcomes also indicated a significant negative impact on the processing of B-cell epitopes, leading to a compromised humoral response to *E. tenella*. This study revealed that specific B-cell epitope responses varied by immunizing dose, highlighting the complex interactions between the host immune system and the pathogen. The insights gained through the current analysis of specific B-cell epitope response can contribute to developing targeted vaccines capable of eliciting a robust and protective immune response [34,40,45,47].

Immunoblots of both asexual zoite stages using antisera from the 5K group revealed more antigens than previously described [18,41,44,46]. However, certain immunodominant antigens exhibit molecular weights that are different from those previously described. For example, this study did not detect the 44–45 kDa antigen identified with chicken and rabbit antisera in sporulated oocysts or sporozoites of *E. tenella* [18,34,44,46]. We only observed bands with molecular weights close to 42 and 47 kDa. Otherwise, Constantinoiu et al. [39] identified a predominant antigen at 31 kDa in sporozoites and another at 34 kDa in merozoites during a priming/challenge test with *E. tenella*, which corresponds closely to the two bands that we observed in both antigen preparations. The antigens recognized by antibodies against *Eimeria* sp. may change over time due to several aforementioned factors, which could account for the slight discrepancies in antigen recognition described in previous investigations compared to the antigens observed in our study [18,31,44,45,46].

When every antiserum from the pullets of the 5K group was probed individually in immunoblots, the antiserum from Pullet 1 showed the most distinct antigen recognition pattern for both life-cycle stages of *E. tenella* (Figure 9). In contrast, Pullets 2 and 3 displayed barely detectable bands on both asexual zoite stages. However, all birds displayed a dominant band of ~26 kDa. This polypeptide has been reported as immunodominant and protective in early studies by Karkhanis et al. [48]. According to recent findings by Liu et al. [45], this antigen may correspond to SAG10, a key surface antigen found in several *E. tenella* stages. On the other hand, a band with an experimental molecular weight of 26.86 kDa has previously been linked to the sporozoite antigen TA4 precursor (SAG1) [40]. According to several studies, the microneme protein precursor 2 (EtMIC2) fusion protein encodes a polypeptide of 489 amino acids with a predicted molecular mass of 54.8 kDa [39,40]. Using a MALDI-TOF assay, de Venevelles et al. [40] identified EtMIC2 with an experimental molecular weight of 53.4 kDa. Every pullet’s antiserum reacted with a specific band of 53 kDa on *E. tenella* sporozoite and 2nd-generation merozoite antigens, which closely matched the previously estimated size of EtMIC2 [39]. Each pullet antiserum recognized a band of ~105 kDa on both life-cycle stages of *E. tenella*. Previous research by de Venevelles et al. [40] suggested that a polypeptide with a similar molecular mass is closely related to EtMIC1. The mean OD per group was used to assess the reactogenicity in the ELISA test against both asexual zoite stages of *E. tenella*. The uniform response to both antigens suggests that all birds had developed immunity to coccidia following the last booster [29,31,34] (Figure 5). However, individual analysis of each antiserum from the 5K-immunized group revealed varying reactions against both stages of the *E. tenella* life cycle [13,31,34] (Figure 8). Pullets immunized with 5K oocysts exhibited a strong individual correlation between the intensity of antigen recognition in Western blotting and their respective reactogenicity in ELISA. Pullet 1 showed the highest titer, while Pullet 2 displayed the lowest (Figure 8). Constantinoiu et al. [31] observed a similar antigen recognition pattern for *E. tenella* sporozoites and the 2nd generation of merozoites in the antiserums of five inbred broiler chickens. The uniform response observed in the inbred broiler chickens contrasts with the varied humoral response and antigen recognition patterns displayed by our three hybrid pullets. However, considering the homogeneous recognition of certain polypeptides as previously described, it is apparent that specific antigens of *E. tenella* exert a significant influence, eliciting a stronger immune response, but the response appears to be polypeptide-specific regardless of the host genotype background [2,4,8,29,31,47].

In the current study, the methodology used was enough to identify significant differences in humoral responses of different primary immunizing doses in pullet hens; however, to enhance understanding about relationship between different dosages of *Eimeria* sp. infection and intestinal integrity of the GALT is critical analyze the inflammatory immune response exhaustively. Specific expressions of some inflammatory cytokines, such as IFN-γ and IL-10, have been used to study the inflammatory immune response found during *Eimeria* infections [49,50]. Recently, cytokine transcription has been used as an indicator of the inflammatory immune response [15,17,19]. However, the results are not yet good enough regarding statistical accuracy; moreover, future improvements in this methodology could facilitate its use as a standard indicator in preclinical and clinical *Eimeria* sp. challenge models. Further investigations exploring the inflammatory immune response, such as pro- and anti-inflammatory cytokine transcription, T-cell-mediated immunohistochemistry, oxidative status, tight junction protein gene expression, and histomorphology changes at GIT, would offer a more comprehensive understanding of the interaction between the host and the parasite [2,4,9,11,14,15,16,17,19,34].

The immunoglobulins Y from Group 5K could facilitate the identification of several immunodominant B-cell antigens of *E. tenella*, such as EtSAG1 (TA4), EtSAG10, EtMIC1, and EtMIC2. These proteins can be sequenced, followed by cloning of their corresponding cDNA, enabling the production of novel recombinant or subunit vaccines. Indeed, these next-generation vaccines could help to design suitable vaccination strategies to mitigate the adverse effects of avian coccidia field outbreaks.

## 5. Conclusions

The results demonstrate that different immunizing doses of *E. tenella* significantly impact the total oocyst output, which suggests a dose-dependent relationship between the degree of parasite exposure and the level of infection. The primary immunization of hybrid pullets with a dose of 5000 *E. tenella* oocysts/bird induces significant oocyst production and contributes to the development of sterilizing immunity. This study has revealed that specific B-cell antigen responses varied according to the size of the primary immunizing dose. Understanding the relationship between the early immunizing dose and oocyst output can aid in designing adequate vaccination schedules to minimize parasitic burden and improve flock health. This study serves as a steppingstone towards unravelling the complexities of *E. tenella* infections in hybrid pullets. Elucidating the impact of an immunizing dose on oocyst output and specific B-cell antigen response, it contributes to the body of knowledge concerning host immunity to this important poultry pathogen. Ultimately, these insights can guide the development of more effective control measures and contribute to improving poultry health and welfare.

## Data Availability

This study’s original contributions are included in the article, and further inquiries can be directed to the corresponding authors.
